# 

**Published:** 1900-09

**Authors:** 


### CONTENTS.

ORIGINAL COMMUNICATIONS. PAGE

The Extraction of Living Pulps from Teeth under the Influence of Cocaine Anæsthesia produced with the Aid of Mechanical Pressure. By WILSON ZERFING, D.D.S., Philadelphia . . . 581
Some Methods of extirpating Pulps, and Subsequent Treatment. By DR. JAMES B. LOCHERTY . . . 590
Dental Work among the Poor: How can it best be accomplished? By H. A. KELLEY, D.M.D., Portland, Me. . . . 597
As We see It. By DR. G. ALDEN MILLS, New York . . . 609

ABSTRACTS AND TRANSLATIONS.

Cocaine and its Rational Antidote. By G. LENOX CURTIS, M.D., New York City 612

REPORTS OF SOCIETY MEETINGS.

The New York Institute of Stomatology . . . 615

EDITORIAL.

The Present and Future of State Boards . . . 630

OBITUARY.

E. Henry Neall, D.D.S. . . . 636
Resolutions of Respect to Dr. Theodore Menges . . . 637

DOMESTIC CORRESPONDENCE.

The New Method of inducing Sleep without Drugs.—One Hundred Dollar Prize . 638

NOTES AND COMMENTS . . . 639

CURRENT NEWS.

Harvard Dental Alumni Association . . . 641
New Dental Society . . . 641
National Dental Association . . . 642
Vermont Board of Dental Examiners . . . 642
Missouri State Dental Association . . . 643
Harvard Odontological Society . . . 643
First District Dental Society of Illinois . . . 644
Northeastern Dental Association . . . 644

